# Superb electromagnetic wave-absorbing composites based on large-scale graphene and carbon nanotube films

**DOI:** 10.1038/s41598-017-02639-7

**Published:** 2017-05-24

**Authors:** Jinsong Li, Weibang Lu, Jonghwan Suhr, Hang Chen, John Q. Xiao, Tsu-Wei Chou

**Affiliations:** 1 School of Physics and Nuclear Energy Engineering, Beihang University, Beijing, 100191 China; 2 Department of Mechanical Engineering, University of Delaware, Newark, DE 19716 United States; 3 Suzhou Institute of Nano-Tech and Nano-Bionics, Chinese Academy of Sciences, Suzhou, 215123 China; 4 Department of Polymer Science and Engineering, Sungkyunkwan University, Suwon, 440-746 Republic of Korea; 5 Department of Physics and Astronomy, University of Delaware, Newark, DE 19716 United States

## Abstract

Graphene has sparked extensive research interest for its excellent physical properties and its unique potential for application in absorption of electromagnetic waves. However, the processing of stable large-scale graphene and magnetic particles on a micrometer-thick conductive support is a formidable challenge for achieving high reflection loss and impedance matching between the absorber and free space. Herein, a novel and simple approach for the processing of a CNT film-Fe_3_O_4_-large scale graphene composite is studied. The Fe_3_O_4_ particles with size in the range of 20–200 nm are uniformly aligned along the axial direction of the CNTs. The composite exhibits exceptionally high wave absorption capacity even at a very low thickness. Minimum reflection loss of −44.7 dB and absorbing bandwidth of 4.7 GHz at −10 dB are achieved in composites with one-layer graphene in six-layer CNT film-Fe_3_O_4_ prepared from 0.04 M FeCl_3_. Microstructural and theoretical studies of the wave-absorbing mechanism reveal a unique Debye dipolar relaxation with an Eddy current effect in the absorbing bandwidth.

## Introduction

The rapid development of modern electronic equipment and wireless devices has resulted in severe electromagnetic (EM) radiation pollution, which has implications in human health and the normal functioning of electronics. Thus, the design and exploration of effective EM wave absorption materials is of basic scientific importance^1–3^. EM waves consist of a time-varying electric and magnetic field, and EM wave absorption is achieved by the attenuation of EM wave energy through dielectric loss and magnetic loss as well as electromagnetic impedance match, which can minimize the reflection of the incident EM wave^4^. Therefore, dielectric loss and magnetic loss are the main factors determining absorption capability. The dielectric tangent loss (tan δ_ε_ = ε″/ε′) is proportional to the dielectric loss, and it describes the dissipation of energy supplied by an external electric field as motion and heat^5^. Magnetic tangent loss (tan δ_μ_ = μ″/μ′) is known as magnetic loss factor or power factor; it is a measure of the energy loss inside a magnetic material due to the phase delay between the applied and induced magnetic fields.

Good impedance matching between the specimen and free space is necessary for enhancing EM wave absorption capability. The impedance matching coefficient |ZinZ0-1| can be expressed as^6^

1 $$|{Z}_{{\rm{in}}}/{Z}_{0}|={({\mu }_{r}/{\varepsilon }_{r})}^{1/2}\,\tanh \,[j(2\pi fd/c){({\mu }_{r}{\varepsilon }_{r})}^{1/2}]$$

And the reflection loss (RL) value is calculated according to the transmission line theory by Equation (2):

2 $${\rm{RL}}({\rm{dB}})=20\,\mathrm{log}\,|({Z}_{{\rm{in}}}-{Z}_{{\rm{0}}})/({Z}_{{\rm{in}}}+{Z}_{{\rm{0}}})|$$

Here, Z in is the input impedance of the absorber, Z_0_ is the characteristic impedance of free space (377 Ω)^7^. ε_r_ = ε′ − jε″ and μ_r_ = μ′ − jμ″ are, respectively, the complex permittivity and permeability of the absorber, c is the velocity of the EM wave in free space, f is the frequency of the EM wave, and d denotes the absorber thickness.

Carbon nanotubes (CNTs) offer the unique potential to be excellent EM wave absorbents. Besides their superior mechanical and thermal properties^8^, the carrier mobility and current carrying capacity of CNTs is up to 10^5^ cm^2^ V^−1^ s^−1^ and 10^9^ A cm^−1^, respectively^9^. Most of the research effort to date has focused on CNTs decorated with magnetic metal or metal oxide particles for enhancing magnetic attenuation. However, the anticipated improvement in EM wave absorption performance has often been hindered by the poor dispersion of CNTs and their impedance mismatch with free space^10^. CNT films, on the other hand, not only retain the high permittivity of individual CNTs in an isotropic network structure but also greatly facilitate the dispersion of CNTs in the matrix^11^. Furthermore, the dispersion of Fe_3_O_4_ nanoparticles on the surface of CNTs can effectively improve permeability and impedance matching^12^. Thus, the CNT film-Fe_3_O_4_ absorber provides a highly desirable combination to enhance dielectric and magnetic loss and impedance matching as well as facilitate ease in composites processing.

The other focus of this research is on graphene, which is well known for its excellent electronic, chemical and physical properties^13–15^. Graphene can support current densities greater than 10^8^ A cm^−2^ ^16^, its carrier mobility can exceed 15,000 cm^2^ V^−1^ s^−1^ even under ambient conditions^17^, and its real and imaginary parts of the theoretical permittivity exceed 10^4^ at frequency less than 400 GHz^18^. Theoretical studies of large-scale graphene layers have shown strong EM wave absorption for normal incident wave. The maximum absorption reaches 50 percent at six layers of graphene and one-third of the incident EM energy is dissipated at a graphene-polymer interface^19, 20^. Although large-scale multilayer graphene/methyl methacrylate for EM wave absorption has been suggested theoretically and realized in numerical simulations^18–20^, large-scale graphene absorption has never been demonstrated experimentally. This is due to the fact that large-scale graphene suffers from statistical fluctuations in strength and toughness as well as impedance mismatch with free space due to the high permittivity and negligible permeability^21^. Consequently, almost all published work so far on graphene-related EM-absorbing materials has focused on graphene powder; little research has been devoted to the EM wave-absorbing performance of large-scale graphene. Moreover, in order to enhance impedance matching and absorption of both electric and magnetic fields in graphene, the improved decoration and blend of magnetic metal or metal oxide particles have become effective approaches for researchers. Some reports demonstrated that the combination of Fe_3_O_4_^22–25^, TiO_2_^26^ and NiFe_2_O_4_^27^ magnetic nanoparticles with reduced graphene oxide powders improved the EM wave-absorbing property. However, the magnetic nanoparticles supported on graphene particles are generally aggregated, resulting in uneven distribution^25^. Therefore, the design and processing of high-performance EM-absorbing materials based on CNT and graphene remain a formidable challenge.

Here, a novel and simple approach was adopted to fabricate CNT film-Fe_3_O_4_ composites by the solvothermal method. The Fe_3_O_4_ nanoparticles on the surface of CNTs not only improved permeability but also enhanced impedance matching of the CNT film-Fe_3_O_4_-graphene composite. Then, the CNT film-Fe_3_O_4_ was used as a support substrate to prepare a large-scale graphene (LSG)-CNT film-Fe_3_O_4_ composite, which not only retained the dielectric performance of CNT and graphene but also avoided their aggregation.

## Results

To enhance the ability of EM wave absorption, highly conductive films (thickness: 2 μm; density: 807 mg cm^−3^) with randomly oriented CNTs coated by Fe_3_O_4_ nanoparticles were used as absorbing layers, the single chemical vapor deposition graphene were laid on the surface of CNT-Fe_3_O_4_ film as blocking layer (Supplementary Fig. S1), and the CNT film-Fe_3_O_4_ with epoxy resin (EPON^TM^ Resin 862) was impedance matching layer. A schematic illustration of the fabrication process for constructing CNT film-Fe_3_O_4_-graphene-epoxy composites can be found as Supplementary Fig. S2.

**Figure 1:**
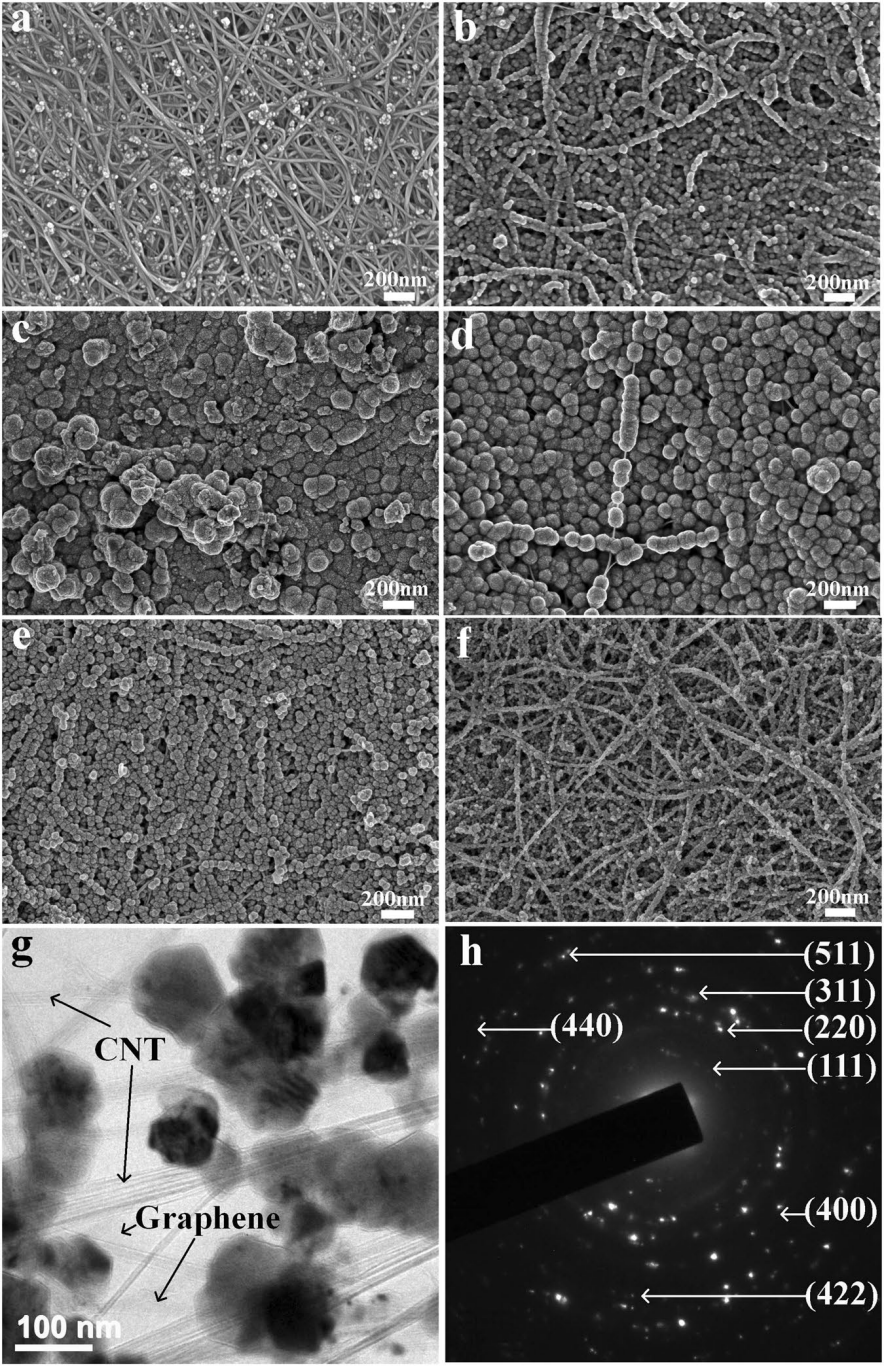
Scanning electron microscope images of the composite prepared at FeCl_3_ concentrations of 0.01–0.10 M: (**a**) 0.01 M, (**b**) 0.02 M, (**c**) 0.03 M, (**d**) 0.04 M, (**e**) 0.05 M and (**f**) 0.10 M. (**g**) Transmission electron microscopy of CNT film-Fe_3_O_4_-0.04 M-graphene. (**h**) The selected-area electron diffraction pattern of Fe_3_O_4_ nanoparticle.

The CNT film-Fe_3_O_4_ composites were processed using the solvothermal method. The morphology and diameters of the Fe_3_O_4_ particles prepared with different FeCl_3_ concentrations are shown in Fig. 1. For the CNT film-Fe_3_O_4_ composite prepared from 0.01 M FeCl_3_, Fe_3_O_4_ nanocrystals cover the CNT surface and form a few spherical particles with diameters ranging from 10–20 nm (Fig. 1a). The Fe_3_O_4_ particles of the as-prepared CNT film-Fe_3_O_4_ composite at FeCl_3_ concentrations of 0.02–0.10 M are spherical with a uniform diameter, aligned along the axial direction of the CNTs. It should be noted that with the increase of FeCl_3_ concentration from 0.02 to 0.10 M, the diameters of the Fe_3_O_4_ particles increase to a maximum value of 200 nm at 0.03 M and then decrease with a minimum value of 20–30 nm at 0.10 M (Fig. 1b–f). Such observations provide clear evidence of the direct relationship between Fe_3_O_4_ nanoparticle size and FeCl_3_ concentration. The growth of the Fe_3_O_4_ nanoparticles follows the two-stage growth model, in which nanocrystals nucleate first and then aggregate into larger particles on the CNT surface^28^.

**Figure 2:**
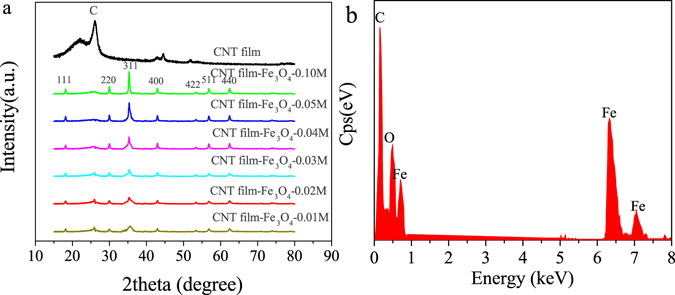
(**a**) XRD patterns of CNT film-Fe_3_O_4_ composite. (**b**) EDS pattern of CNT film-Fe_3_O_4_-0.05 M composite.

Figure 1g is a TEM image of a CNT film-Fe_3_O_4_-0.04 M graphene composite. Crystal planes are identified in the selected-area electron diffraction pattern (Fig. 1h), which is consistent with the XRD pattern. Figure 2a shows the XRD patterns of the pure CNT film and CNT film-Fe_3_O_4_ composites prepared at various FeCl_3_ concentrations. The peak positions and relative intensities match well with the standard XRD data (JCPDS no. 19–0629), and the seven peaks at 18.17°, 30.07°, 35.48°, 43.01°, 53.42°, 57.04° and 62.55° are associated with the (111), (220), (311), (400), (422), (511) and (440) crystal planes of Fe_3_O_4_, respectively. No additional peaks belonging to other phases are observed, indicating the good crystallinity and high purity of the Fe_3_O_4_ nanoparticles. The energy dispersive spectroscopy (EDS) as shown in Fig. 1b reveals that the CNT film-Fe_3_O_4_–0.05 M composite is mainly composed of C, O, and Fe elements.

The average sizes of nanocrystals in the Fe_3_O_4_ nanoparticles are calculated for the strongest peak (311) by the Debye–Scherrer formula. The nanocrystal sizes from the data of Fig. 2 are 9.7, 14.5, 16.4, 18.7, 22.3 and 27.2 nm for CNT film-Fe_3_O_4_ composites prepared from 0.01, 0.02, 0.03, 0.04, 0.05 and 0.10 M FeCl_3_, respectively; this implies that the nanocrystal size increases significantly with increasing concentration of FeCl_3_. The saturation magnetization and relaxation values are directly related to the size of the nanocrystal size, and with the increase of Fe_3_O_4_ nanocrystal size, both the saturation magnetization and relaxation rate increase^29^.

**Figure 3:**
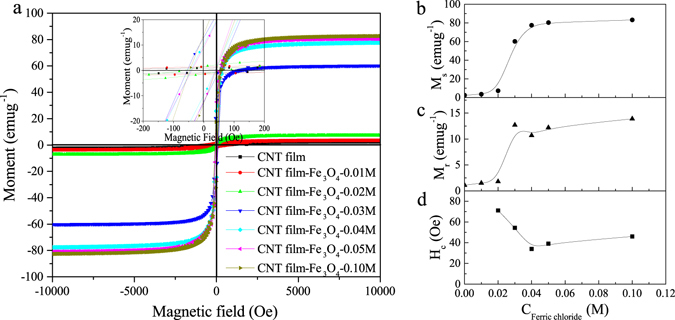
(**a**) Magnetic hysteresis loops of composites at room temperature (inset shows expanded low field hysteresis curves). (**b**) Variation of saturation magnetization with FeCl_3_ concentration. (**c**) Variation of remnant magnetization with FeCl_3_ concentration. (**d**) Variation of coercivity with FeCl_3_ concentration.

The magnetic properties of the CNT film-Fe_3_O_4_ composites at room temperature are shown in Fig. 3. Significant hysteresis loops indicate the ferromagnetic behavior of CNT film-Fe_3_O_4_ composites (Fig. 3a). The saturation magnetization (M_s_) and remnant magnetization (M_r_) of the CNT film-Fe_3_O_4_-0.01 M and 0.02 M remain nearly the same as those of the CNT film. At FeCl_3_ concentrations higher than 0.02 M, the M_s_ and M_r_ values increase rapidly, as shown in Fig. 3b and c. The variation of coercivity (H_c_) with FeCl_3_ concentration is shown in Fig. 3d. The values of μ_r_ and anisotropy energy, which are related to the values of M_s_ and H_c_, affect the EM wave absorption capacities^30^.

**Figure 4:**
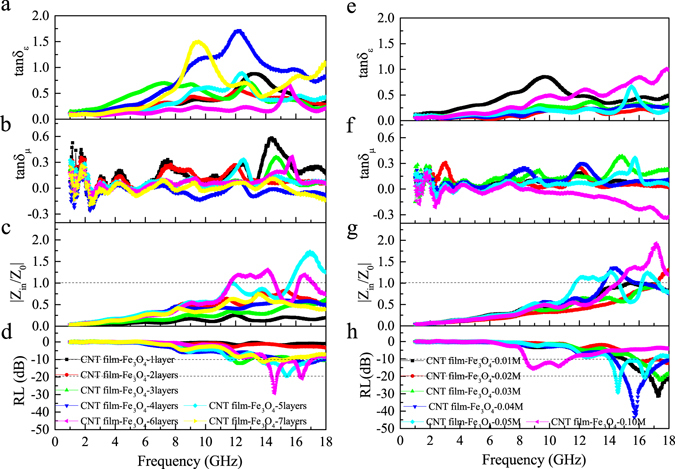
(**a**) Dielectric tangent loss, (**b**) magnetic tangent loss, (**c**) impedance matching coefficient |Z_in_/Z_0_| and (**d**) reflection loss of the CNT film-Fe_3_O_4_ composites with one, two, three, four, five, six and seven layers prepared from 0.05 M FeCl_3_. (**e**) Dielectric tangent loss, (**f**) magnetic tangent loss, (**g**) impedance matching coefficient |Z_in_/Z_0_| and (**h**) reflection loss of the CNT film-Fe_3_O_4_ composites with six layers prepared from 0.01, 0.02, 0.03, 0.04, 0.05 and 0.10 M FeCl_3_.

Based on the above magnetic property data and morphology observations, CNT film-Fe_3_O_4_-0.05 M composites with various numbers of layers were fabricated. The values of tan δ_ε_ and tan δ_μ_, impedance matching coefficient |Z_in_/Z_0_| and reflection loss of composites consisting of one to seven CNT film-Fe_3_O_4_-0.05 M layers were deduced from the measured data of relative complex permittivity ε_r_, permeability μ_r_ and thickness d. The effect of the number of layers on the composite EM wave-absorbing properties was studied. The results of Fig. 4a demonstrate that strong peaks of tan δ_ε_ appear when the frequency is higher than 6 GHz. The locations of peaks shift toward lower frequencies with an increase in the number of layers of CNT film-Fe_3_O_4_ composite. The peak intensity varies with the number of layers and reaches a maximum value of 1.72 at four layers. Figure 4b depicts the variation of tan δ_μ_ of the CNT film-Fe_3_O_4_ composites with frequency and number of layers. Strong peaks of tan δ_μ_ can be seen in the frequency range of 10–17 GHz, and the peak location also shifts toward lower frequencies with increasing number of layers. The peak intensity of tan δ_μ_ decreases from a maximum value of 0.58 at one layer to a minimum value of 0.08 at four layers. It should be noted that the values of tan δ_μ_ become negative in some frequency ranges, indicating that the magnetic energy radiated out from the composites due to the high carrier mobility of CNTs^7^. Overall, the above results demonstrate the dependence of the dielectric and magnetic responses on the number of layers of CNT film-Fe_3_O_4_ composite. In addition, the values of tan δ_ε_ are larger than those of tan δ_ε_ for almost the entire frequency range, suggesting that dielectric loss plays a major role in the intrinsic EM wave absorption.

Results of |Z_in_/Z_0_| and RL calculated from the relative permittivity and permeability data are given in Fig. 4c and d, respectively. When |Z_in_/Z_0_| is close to unity, most of the EM waves can effectively enter the composite and then dissipate as heat. Figure 4c shows that when the number of layers is increased, the value of |Z_in_/Z_0_| of the CNT film-Fe_3_O_4_ composites increases and reaches a maximum value of 1.73 at six layers and then decreases to 0.80 at seven layers. Only the values of |Z_in_/Z_0_| of the CNT film-Fe_3_O_4_ composites with five and six layers are close to unity in the frequency range of 10–17 GHz. Figure 4d shows that for the CNT film-Fe_3_O_4_ composite with five layers, the minimum value of reflection loss is −19.3 dB, and the frequency bandwidth at −10 dB is 3.6 GHz. For the CNT film-Fe_3_O_4_ composite with six layers, the minimum value of reflection loss is −26.6 dB, and the frequency bandwidth at −10 dB is 2.9 GHz. However, although the CNT film-Fe_3_O_4_ composites with one, two, three, four and seven layers have higher dielectric loss, the frequency bandwidth at −10 dB and the magnitude of minimum reflection loss are far less than those of five- and six-layer composites.

Based on the results of CNT film-Fe_3_O_4_ composites with different layers, CNT film-Fe_3_O_4_ composites of six layers with different FeCl_3_ concentrations were prepared. As shown in Fig. 4e and f, the values of tan δ_ε_ of the CNT film-Fe_3_O_4_ composites of 0.02–0.05 M are all less than that of the CNT film-Fe_3_O_4_–0.01 M composite, and the values of tan δ_μ_ are all higher than that of the CNT film-Fe_3_O_4_-0.01 M composite. It is interesting that tan δ_ε_ of the CNT film-Fe_3_O_4_-0.10 M increases almost monotonically with frequency. The tan δ_μ_ value of the CNT film-Fe_3_O_4_-0.10 M is negative and shows a decreasing trend at frequencies higher than 8.5 GHz (Fig. 4f). Figure 4g shows that the |Z_in_/Z_0_| values of some composites are close to unity at frequencies higher than 11.7 GHz. The minimum values of reflection loss of the CNT film-Fe_3_O_4_-0.01, 0.04 and 0.05 M composites are -32.0 dB at 17.1 GHz, −43.6 dB at 15.7 GHz and −26.6 dB at 14.5 GHz, respectively, obviously shifting to lower frequency range with increase in FeCl_3_ concentrations (Fig. 4h). For the same composites, the bandwidths of the reflection loss at −10 dB are 3.1 GHz, 3.7 GHz and 2.9 GHz, respectively; their bandwidths of reflection loss at −20 dB are 1.2 GHz, 1.5 GHz and 0.3 GHz, respectively. Although the CNT film-Fe_3_O_4_-0.10 M composite has no strong RL peak, the bandwidth at −10 dB is 2.9 GHz.

**Figure 5:**
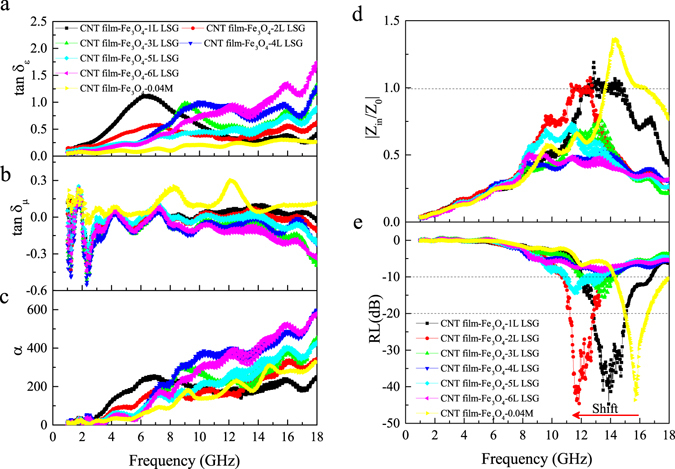
(**a**) Dielectric tangent loss, (**b**) magnetic tangent loss, (**c**) attenuation constant, (**d**) impedance matching coefficient |Z_in_/Z_0_| and (**e**) reflection loss of the CNT film-Fe_3_O_4_-0.04 M-LSG composites with one, two, three, four, five and six layers of graphene.

On the basis of the results summarized above, CNT film-Fe_3_O_4_-LSG composites with six layers of CNT film-Fe_3_O_4_-0.04 M and various numbers of graphene layers have been processed and studied. Although the minimum reflection loss and thickness of the CNT film-Fe_3_O_4_ composite established from the above experiments are highly encouraging, the absorption bandwidth of 3.7 GHz is still relatively small. To further improve the EM wave-absorption performance, it is desirable to pursue a CNT film-Fe_3_O_4_-graphene composite in which the graphene layers are separated from one another by a thin layer of epoxy. Figure 5a and b show that tan δ_ε_ increases and tan δ_μ_ decreases to negative values with an increasing number of graphene layers; the ranges of tan δ_ε_ and tan δ_μ_ are, respectively, 0.2–1.6 and −0.4–0.1 for the frequencies of 3–18 GHz, indicating that the dielectric loss plays a major role in EM wave absorption.

In addition, EM wave attenuation in an absorber is a key factor for achieving EM wave absorption. The EM wave attenuation ability of an absorber can be characterized by the attenuation constant^30^

3 $${\rm{\alpha }}=\sqrt{2}\pi f{c}^{-1}\sqrt{({\rm{\varepsilon }}^{\prime\prime} {\rm{\mu }}^{\prime\prime} -{\rm{\varepsilon }}^{\prime} {\rm{\mu }}^{\prime} )+\sqrt{{({\rm{\varepsilon }}^{\prime\prime} {\rm{\mu }}^{\prime\prime} -{\rm{\varepsilon }}^{\prime} {\rm{\mu }}^{\prime} )}^{2}+{({\rm{\varepsilon }}^{\prime} {\rm{\mu }}^{\prime\prime} -{\rm{\varepsilon }}^{\prime\prime} {\rm{\mu }}^{\prime} )}^{2}}}$$

The results of Fig. 5c show that all samples exhibit a high attenuation constant (larger than 150) in the frequency range of 7–18 GHz. The values of α increased with an increasing number of graphene layers, and an obvious peak appears when the number of graphene layers is less than four. The locations of peaks shift toward higher frequencies as the number of graphene layers increases. Figure 5d shows the variations of impedance matching coefficient |Z_in_/Z_0_| with frequency. The |Z_in_/Z_0_| values of CNT film-Fe_3_O_4_–1L and 2L LSG composites are close to unity in the frequencies of 11–15 GHz, and the corresponding impedance matching bandwidth of frequency is enhanced by the large-scale graphene compared to that of the CNT film-Fe_3_O_4_-0.04 M composite without LSG. However, the |Z_in_/Z_0_| values decrease with the increase of graphene layers.

As shown in Fig. 5e, for the CNT film-Fe_3_O_4_-0.04 M-1L LSG composite, the reflection loss is below −10 dB at 12.0–16.7 GHz, and the minimum value is −44.7 dB at 13.9 GHz; the bandwidths of the reflection loss at −10 dB and −20 dB are 4.7 GHz and 2.2 GHz, respectively. Compared to the CNT film-Fe_3_O_4_-0.04 M composite without graphene, the bandwidths of the reflection loss at −10 dB and −20 dB increase by 27.0 percent and 46.7 percent, respectively; the minimum reflection loss increases by 2.5 percent, and the peak of the reflection loss shifts from 15.7 GHz to 13.9 GHz. For the case of two graphene layers, the reflection loss is below −10 dB at 10.9–13.5 GHz, and the minimum value is −44.6 dB at 11.9 GHz; the bandwidths of the reflection loss at −10 dB and −20 dB are 2.6 GHz and 1.6 GHz, respectively. However, with the further increase of graphene from three to six layers, the absorbing performance deteriorates sharply; the reflection loss has no bandwidth under −20 dB, and there is only a narrow bandwidth at −10 dB.

## Discussion

**Figure 6:**
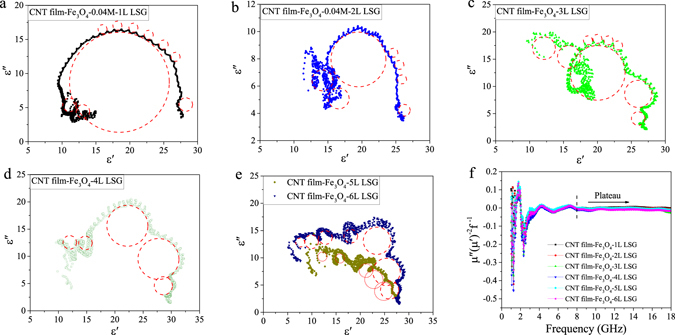
Dielectric Cole-Cole semicircles of the CNT film-Fe_3_O_4_-0.04 M-LSG composites: (**a**) one, (**b**) two, (**c**) three, (**d**) four, (**e**) five and (**f**) six layers of graphene, respectively. (**f**) μ″(μ′)^−2^f^−1^ vs. frequency f for the CNT film-Fe_3_O_4_-0.04 M-LSG composites.

An important mechanism of dielectric loss, which arises from the polarization and its associated relaxation, can be evaluated by the Cole–Cole semicircle (ε″ versus ε′), and each semicircle is correlated with one Debye relaxation process^7^. The Cole–Cole semicircles of the composite samples are shown in Fig. 6a–e. For the CNT film-Fe_3_O_4_-1L LSG composite, one large Cole-Cole semicircle, which consists of many tiny semicircles, and three small semicircles can be identified in Fig. 6a. With an increasing number of graphene layers, the number of Cole−Cole semicircles increases (Fig. 6b–e), the large semicircle decreases in size, and the tiny semicircles increase in size. The presence of semicircles in the ε″ ~ ε′ relation provides the evidence that there are dual dielectric relaxation processes in the CNT film-Fe_3_O_4_-LSG composites and demonstrates that large-scale graphene improves the intensity of the Debye dipolar relaxation process^31^.

In the microwave frequency band, magnetic loss mainly comes from the eddy current effect, along with natural and exchange resonance^6^. The eddy current loss contribution to the imaginary part of permeability is related to the permeability of vacuum (μ_0_), thickness (d) and electrical conductivity (σ) of the composite as μ″ = 2πμ_0_(μ′)^2^σd^2^f/3. If the magnetic loss results from the eddy current effect, μ″(μ′)^−2^f^−1^ should be constant when the frequency varies. It is shown in Fig. 6f that the values of μ″(μ′)^−2^f^−1^ drastically fluctuate in the frequency range of 2.0–8.0 GHz. However, when f >8.0 GHz, the values of μ″(μ′)^−2^f^−1^ remain essentially constant. Thus, it can be concluded that the magnetic loss at 2.0–8.0 GHz is caused by natural resonance, and the other peaks at 8.0–18.0 GHz are ascribed to the eddy current effect.

**Figure 7:**
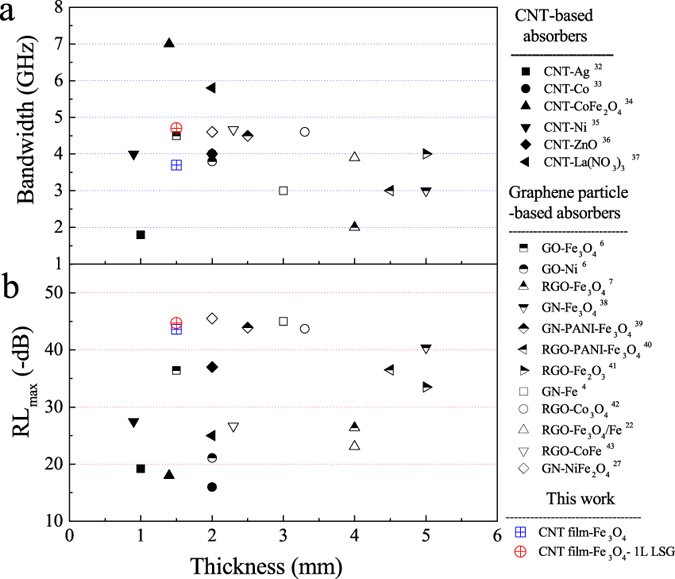
Maximum of reflection loss, absorbing bandwidth and thickness of this work compared with those of previously reported graphene- and CNT-based wave-absorbing composites: (**a**) absorbing bandwidth at −10 dB, (**b**) maximum of reflection loss. (GN: graphene; GO: graphene oxide; RGO: reduced graphene oxide^4, 6, 7, 22, 27, 35–46^.

A superb EM wave-absorbing material is expected to possess not only a high RL factor and broad absorption bandwidth, but also a fine thickness and, hence, light weight. The reflection loss, absorption bandwidth and thickness achieved in the composites of this work are shown in Fig. 7 and compared with those of previously reported graphene- and CNT-based EM wave-absorbing composites. Compared to other graphene particle or CNT-based absorbers, the absorption bandwidth achieved in this work is 0.6–161.1 percent higher than the others except for the CNT-CoFe_2_O_4_ and CNT-La(NO_3_)_3_ absorbers (Fig. 7a); the composite thickness of this work is reduced by 25–70 percent except for the CNT-Ni, -Ag and -La(NO_3_)_3_ absorbers at 0.9, 1 and 1.4 mm, respectively, and the GO-Fe_3_O_4_ absorber also at 1.5 mm. However, the maximum reflection loss of this work is 1.2–2.5 times the values of the above five absorbers CNT-Ni, -Ag -CoFe_2_O_4_ and -La(NO_3_)_3_, GO-Fe_3_O_4_ (Fig. 7b).

In summary, we have demonstrated that composites composed of large-scale graphene and CNT film-Fe_3_O_4_ substrate demonstrate superb EM wave-absorbing performance. Through optimization of the number of layers of CNT film-Fe_3_O_4_ composite and FeCl_3_ concentrations, the CNT film-Fe_3_O_4_-LSG-epoxy composites are processed using six layers of CNT film-Fe_3_O_4_ prepared from 0.04 M FeCl_3_. The CNT film-Fe_3_O_4_-1L LSG-epoxy composite has the highest wave-absorbing capability. Increasing the number of layers of graphene leads to an increase in wave attenuation ability but a decrease in impedance matching coefficient and a corresponding decrease in minimum reflection loss and absorbing bandwidth. Compared to the CNT film-Fe_3_O_4_-0.04 M composite without graphene, the minimum reflection loss and absorption bandwidth at −10 dB of the corresponding composite with one-layer graphene increase by 27.0 percent and 2.5 percent, respectively. This study provides a thorough explanation of nanocarbon-based electromagnetic wave-absorbing composites and opens up the opportunity to apply large-scale CNT and graphene films for EM wave absorption.

## Methods

### Sample preparation process

**Figure 8:**
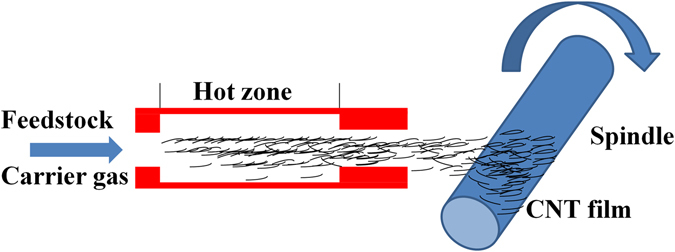
Schematics of CNT film fabrication.

CNT film was fabricated by floating catalyst chemical vapor deposition (FCCVD) method^32^. Figure 8 shows the schematics of CNT film fabrication. In this process, the feedstock of ethanol containing 1.2 vol % ferrocene and 0.4 vol % thiophene, carried by Ar/H_2_, was injected at the rate of 20 mL/h into a reactor at high temperature (~1300 °C). Ethanol, ferrocene, and thiophene were used as carbon source, catalyst precursor, and promotor, respectively. CNTs were synthesized and entangled in the hot zone forming a stocking-like aerogel. The CNT aerogel was then blown out continuously from the reactor and collected layer-by-layer by a wheel rotating perpendicularly to the gas flow, which formed macroscopic CNT film (thickness: 2 μm; density: 807 mg/cm^3^) with randomly oriented CNTs^33^. This CNT film was further densified by spraying ethanol onto it to enhance its mechanical and physical properties.

The single-layer graphene films were synthesized on a Cu substrate using the CVD method. A Cu foil was annealed at 1000 °C for 1.5 h in a quartz tube with a 2 sccm H_2_ flow. Subsequently, a 20 sccm CH_4_ flow was introduced for graphene growth at 30 mTorr for 0.5 h. Then, the furnace was quickly cooled to room temperature under He flow, and the single-layer graphene on Cu foil was obtained^34^. The size of the graphen was 25 mm × 20 mm.

The CNT film-Fe_3_O_4_ composites were prepared in a solvothermal system by the reduction reaction between FeCl_3_ and ethylene glycol in the presence of CNT film. FeCl_3_·6H_2_O was dissolved into 50 mL ethylene glycol followed by the addition of 3.0 NaAc (protective agent) and 10 mL ethylene diamine (EDA) to form a homogeneous solution with magnetic stirring. The mixture was then transferred to a 100 mL Teflon-lined stainless steel autoclave, and 30 cm^2^ CNT film was completely immersed in the solution for solvothermal reaction at 200 °C for 10 h (Supplementary Fig. S3). After washed with deionized water five times and dried under vacuum at 70 °C for 20 min, the prepared production was cut uniformly into six pieces with dimensions 25 mm × 20 mm. The loading Fe_3_O_4_ prepared at FeCl_3_ concentrations of 0.01, 0.2, 0.3, 0.4, 0.5 and 0.10 M were 2, 4, 6, 6, 5 and 3 × 10^−5^ g/cm^2^, respectively. The Cu foil of the PMMA-graphene-copper foil was etched away by 0.2gml^−1^ FeCl_3_. After rinsing with deionized water, the floating PMMA/graphene film was scooped up by CNT film-Fe_3_O_4_ film. Then, the precoated PMMA was dissolved by acetone. Finally, the laminated graphene on CNT film-Fe_3_O_4_ substrate was obtained.

The individual CNT-Fe_3_O_4_-graphene films were first sprayed with a diluted epoxy solution prepared by mixing an epoxy base agent (EPON^TM^ Resin 862), curing agent (DETDA) and ethyl acetate in the ratio of 10:1:100, followed by degassing in a vacuum oven for 30 min. Next, a CNT film-Fe_3_O_4_-graphene-epoxy composite was prepared by laminating the as fabricated films by spin coating Resin 862 and DETDA in the ratio of 10:1, followed by curing at 100 °C for 1 h. The thickness of one layer of CNT film-Fe_3_O_4_-epoxy composite was about 0.25 mm.

### Characterization

X-ray diffraction (XRD) patterns of the composites were measured on a Bruker D8 advance diffractometer using a Cu Kα source (λ = 0.154056 nm). Scanning electron microscopy (SEM) and transmission electron microscope (TEM) images were performed on the Auriga 60 and TEM Talos F200C, respectively. A magnetic study was performed by a vibrating sample magnetometer (VSM, Quantum Design, Versalab). The electromagnetic parameters of the samples were measured using a vector network analyzer (Agilent 8722ES) in the range of 1-18 GHz after a full two-port calibration (Supplementary Fig. S4–S6). The composite was machined into a toroidal shaped sample with an outer diameter of 7.0 mm and inner diameter of 3.04 mm.

### Data Availability

All data generated or analysed during this study are included in this published article (and its Supplementary Information files).

## Electronic supplementary material

Superb electromagnetic wave-absorbing composites based on large-scale graphene and carbon nanotube films
