# Supplementary material for: Superb electromagnetic wave-absorbing composites based on large-scale graphene and carbon nanotube films
Source: Sci Rep. 2017 May 24;7:2349. doi: 10.1038/s41598-017-02639-7 (PMC5443822; doi:10.1038/s41598-017-02639-7)
Supplement: Supplementary file 1 — Superb electromagnetic wave-absorbing composites based on large-scale graphene and carbon nanotube films [file 41598_2017_2639_MOESM1_ESM.pdf]

Superb electromagnetic wave-absorbing composites based on large-scale graphene and carbon nanotube films

Jinsong Li[1,2], Weibang Lu[3], Jonghwan Suhr[4], Hang Chen[5], John Q. Xiao[5] and Tsu-Wei Chou[2★]

[1] School of Physics and Nuclear Energy Engineering, Beihang University, Beijing 100191, China.

[2] Department of Mechanical Engineering, University of Delaware, Newark, DE 19716, United States.

[3] Suzhou Institute of Nano-Tech and Nano-Bionics, Chinese Academy of Sciences, Suzhou 215123, China.

[4] Department of Polymer Science and Engineering, Sungkyunkwan University, Suwon 440-746, Republic of Korea.

[5] Department of Physics and Astronomy, University of Delaware, Newark, DE 19716, United States.

★ Corresponding author email: chou@udel.edu

Supplementary Note 1. SEM images of CNT film and CNT-Fe_3O_4-graphene film.

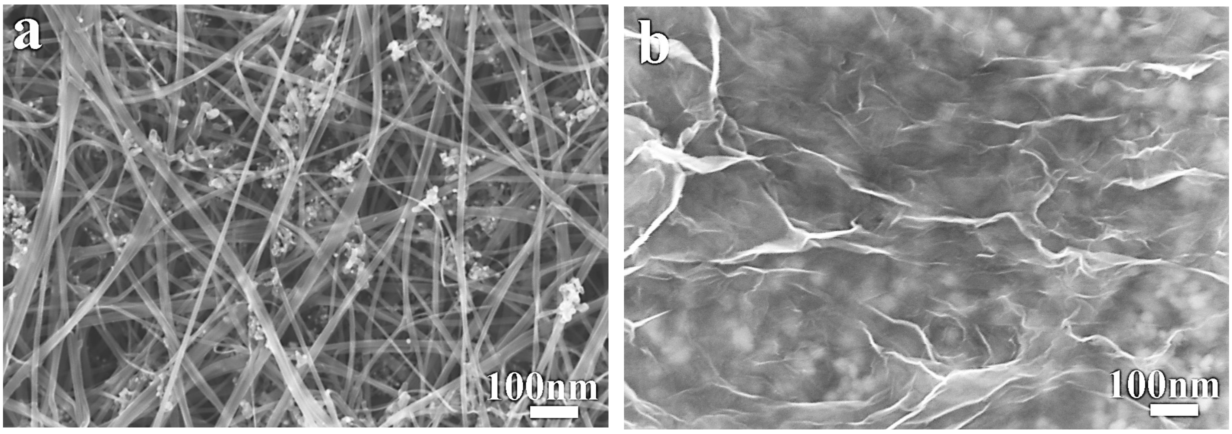

Supplementary Figure 1| (a) SEM image of CNT film. (b) SEM image of CNT-Fe_3O_4-graphene film.

Supplementary Note 2. Process of the CNT film-Fe_3O_4-grpahene composite.

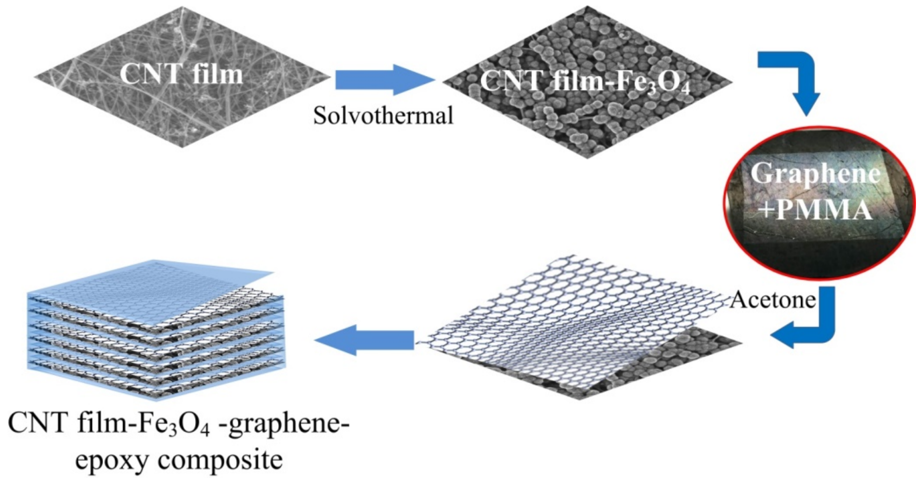

Supplementary Figure 2| Schematic of the process of the CNT film-Fe_3O_4-graphene composite.

Supplementary Note 3. CNT film has good wettability with ethylene glycol, CNT film was completely immersed in the reaction solution.

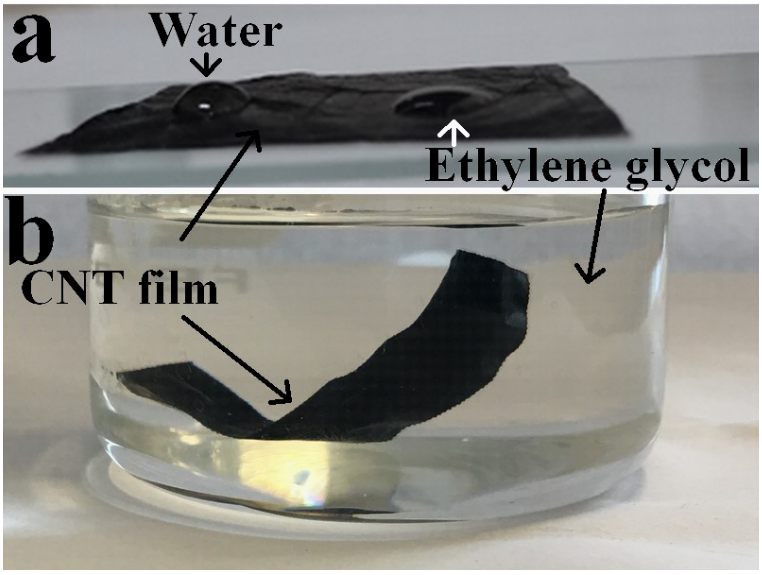

Supplementary Figure 3| Photo images of (a) wettability of CNT film with ethylene glycol and (b) CNT film immersed in ethylene glycol.

Supplementary Note 4. Relative complex permittivity and permeability of the CNT film-Fe_3O_4 composites with one, two, three, four, five, six and seven layers prepared from 0.05 M $FeCl_3$.

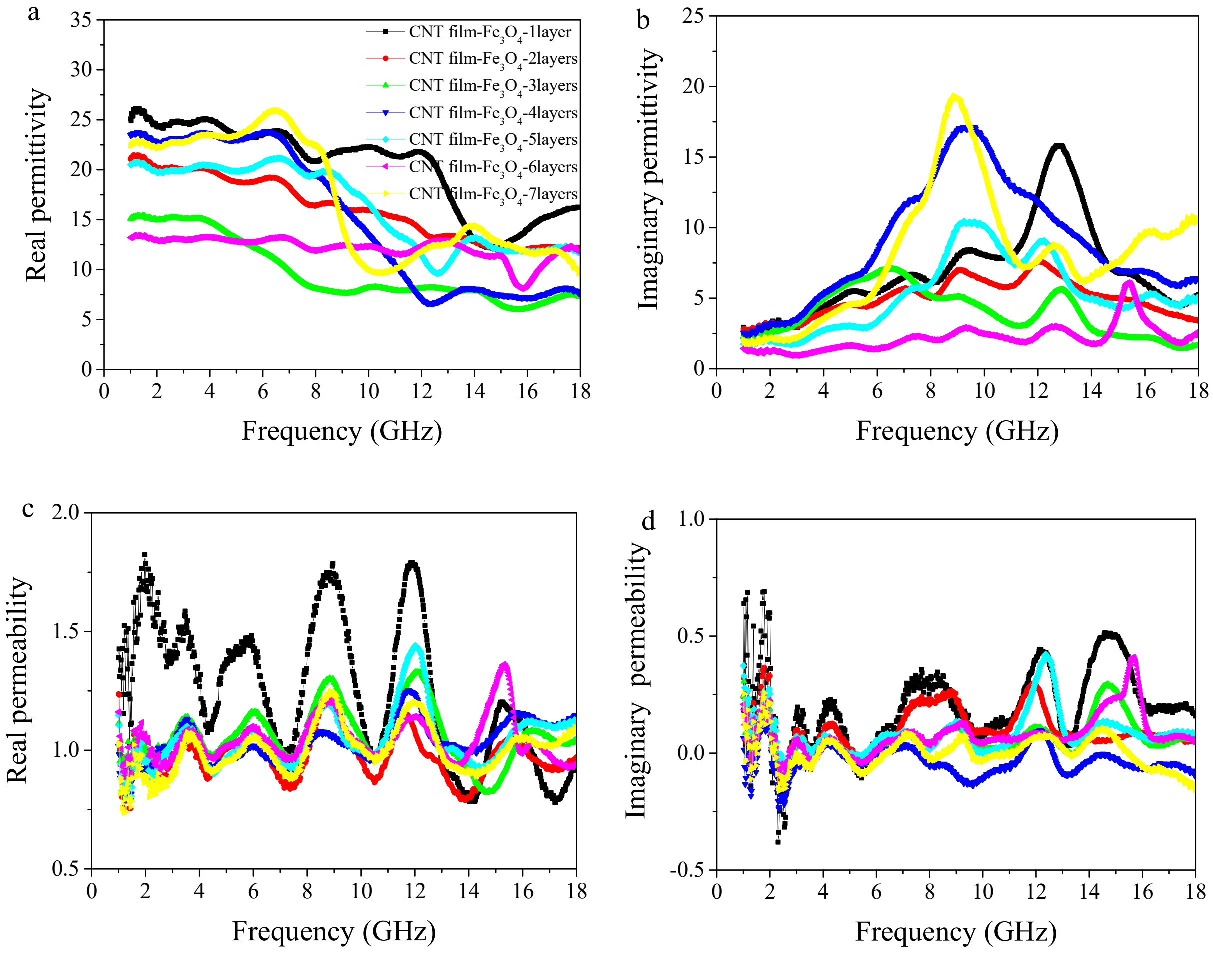

Supplementary Figure 4| (a) real and (b) imaginary parts of the complex permittivity; (c) real and (d) imaginary parts of complex permeability of the CNT film-Fe_3O_4 composites with one, two, three, four, five, six and seven layers prepared from 0.05 M $FeCl_3$.

Supplementary Note 5. Relative complex permittivity and permeability of the CNT film-Fe_3O_4 composites with six-layers prepared from 0.01, 0.02, 0.03, 0.04, 0.05 and 0.10 M $FeCl_3$.

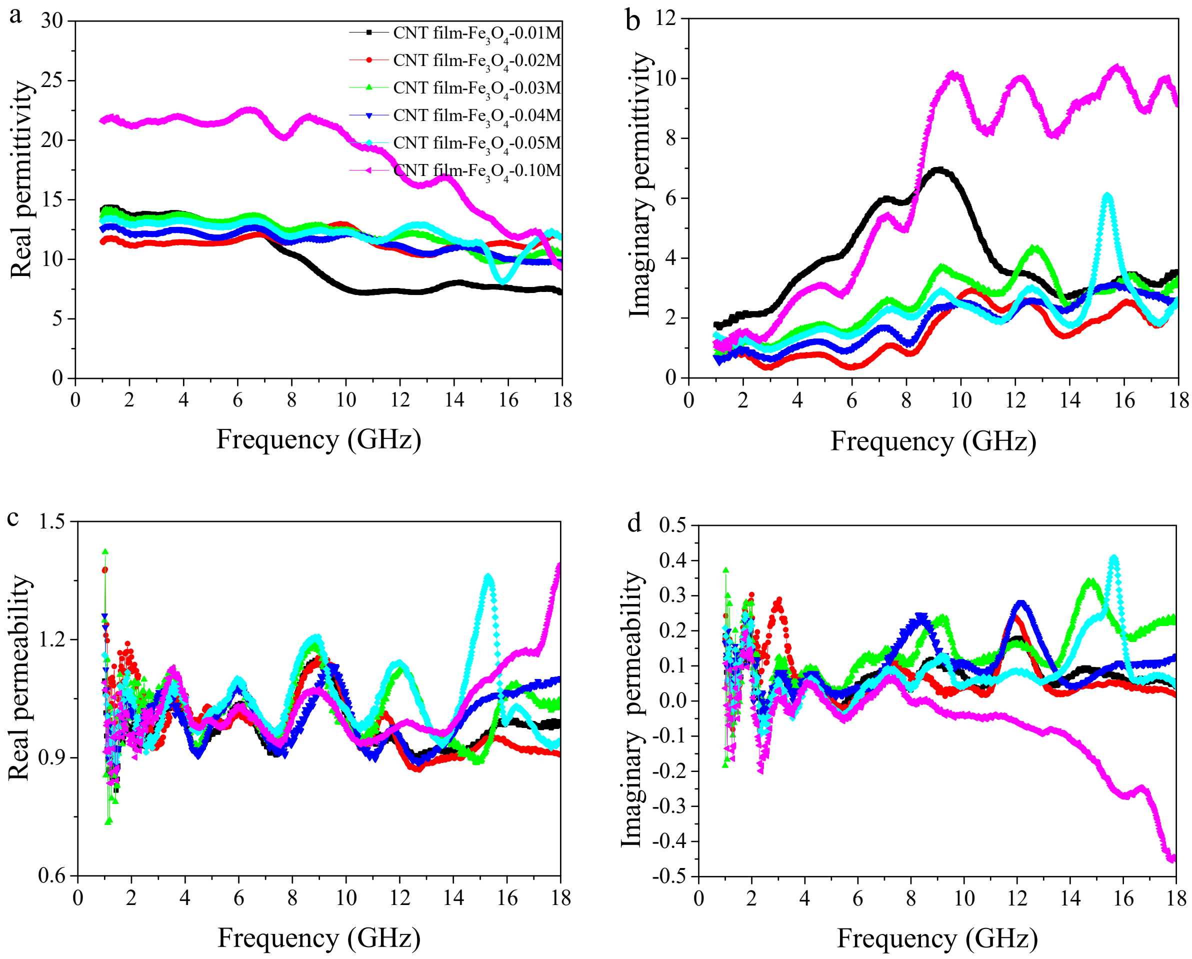

Supplementary Figure 5| (a) real and (b) imaginary parts of the complex permittivity; (c) real and (d) imaginary parts of complex permeability of the CNT film-Fe_3O_4 composites with six-layers prepared from 0.01, 0.02, 0.03, 0.04, 0.05 and 0.10 M $FeCl_3$.

Supplementary Note 6. Relative complex permittivity and permeability of the CNT film-Fe_3O_4 -0.04 M-LSG composites with one, two, three, four, five and six layers of graphene.

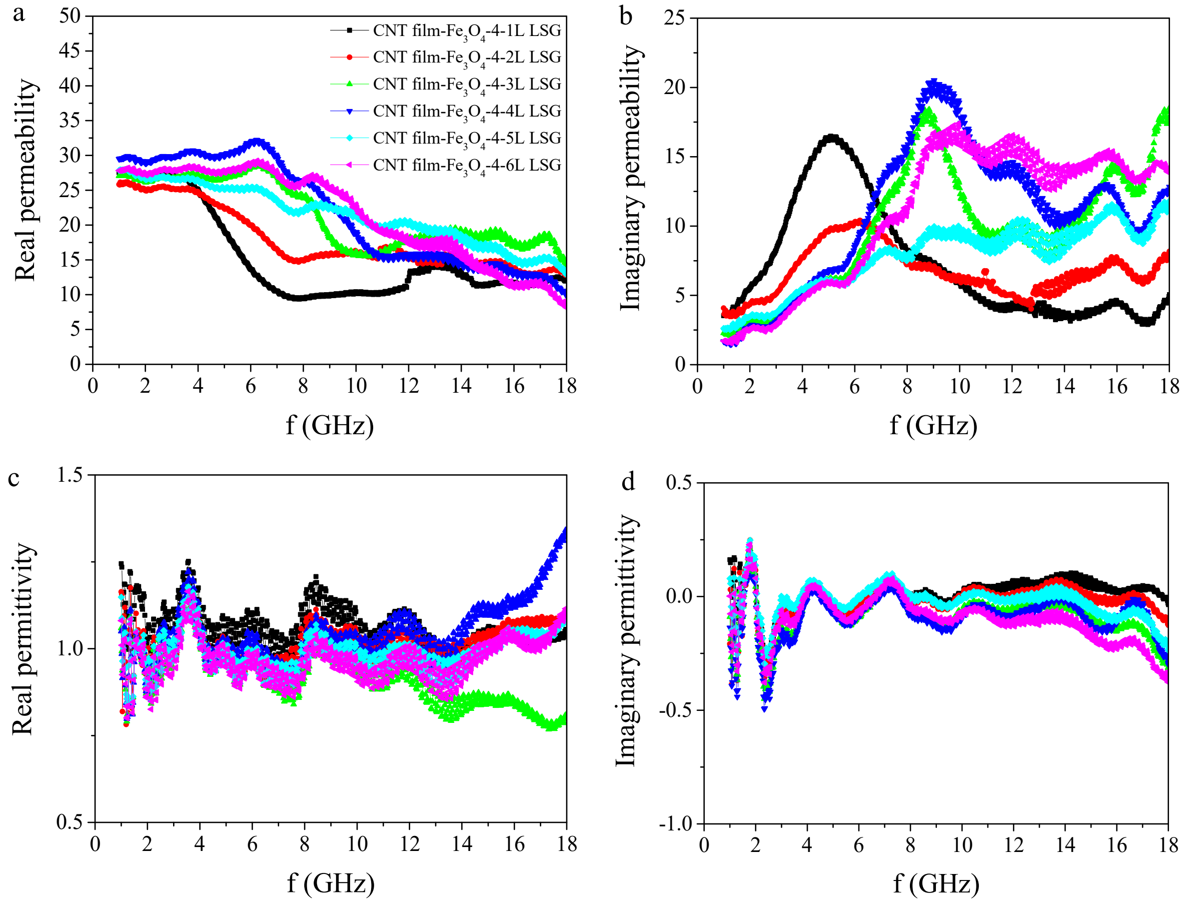

Supplementary Figure 6| (a) real and (b) imaginary parts of the complex permittivity; (c) real and (d) imaginary parts of complex permeability of the CNT film-Fe_3O_4 -0.04 M-LSG composites with one, two, three, four, five and six layers of graphene.
